# Haptic-Optic Fractures in Yamane Flanged Intrascleral Fixated Lenses: A Case Series

**DOI:** 10.18502/jovr.v20.16422

**Published:** 2025-09-02

**Authors:** Rina Su, Paras P. Shah, Elmira Baghdasaryan, Daniel Zhu, Talia Kaden, Jules Winokur, Isha Cheela

**Affiliations:** ^1^Department of Ophthalmology, Northwell Health Eye Institute, Great Neck, NY, USA; ^2^Manhattan Eye, Ear and Throat Hospital, New York, NY, USA

**Keywords:** Haptic-Optic Junction, Intraocular Lens, Scleral Fixation, Yamane

## Abstract

**Purpose:**

To report five cases of structural weakness at the haptic-optic junction of the CT Lucia 602 intraocular lens (IOL) (Carl Zeiss Meditec Inc., Dublin, CA), including two optic breakages and three optic microfractures, while using the Yamane flanged intrascleral haptic fixation surgery (FIHFS).

**Methods:**

A retrospective chart review of five cases that underwent Yamane FIHFS by the same surgeon between 2020 and 2022 was performed. Patients' demographics, operative technique, postoperative outcomes, and adverse events were recorded over a 12-month follow-up period.

**Results:**

One case of optic breakage, noted on postoperative week 1 (POW1), had the optic with the trailing haptic suspended in the vitreous while both haptic flanges remained secured to the sclera, requiring IOL exchange. The second case of optic breakage was noted intraoperatively and required explantation of the IOL with subsequent placement of an iris-fixated IOL. Three cases of optic microfractures were observed with no long-term complications. All five fractures occurred at the junction of the leading haptic.

**Conclusion:**

Optic breakage and microfractures at the haptic-optic junction are unusual complications of intrascleral fixated IOL using the Yamane technique. To the best of our knowledge, this has not been previously reported with the CT Lucia 602 IOL. The authors recommend careful selection of the IOL material for this technique to prevent possible complications.

##  INTRODUCTION

Scleral-fixated intraocular lens (IOL) implantation is one of several options for patients with poor or absent capsular support. While IOLs can be fixated to the sclera using both sutured and sutureless methods, the double needle flanged intrascleral IOL fixation technique – also known as the Yamane technique – has become a frequently used procedure as it avoids the need for sutures, conjunctival peritomies, or scleral flaps.^[1]^ While this surgical method is generally safe and effective, complications may still occur. Here, the authors describe five cases of intraocular optic fractures at the haptic-optic junction of varying severity while using the Yamane flanged intrascleral haptic fixation surgery (FIHFS) technique. Two cases resulted in frank haptic-optic breakage, while three sustained only microfractures in the optic without complete breakages.

##  METHODS

A total of five patients who underwent Yamane FIFHS were included in this study. Initially, the two eyes of two patients with optic breakage during secondary IOL fixation using the Yamane technique were noted between July 1, 2022, and August 31, 2022. This prompted a retrospective review from January 1, 2021, to December 31, 2022, during which an additional three cases of optic microfracture at the haptic-optic junction were identified, none of which resulted in complete breakage. This study was approved by the Institutional Review Board of Northwell Health, NY, USA (IRB approval #21-1135) and conducted in accordance with the tenets of the Declaration of Helsinki. The IRB waived the need for informed consent as patients were de-identified.

A CT Lucia 602 (Carl Zeiss Meditec, Dublin, California, USA) IOL was placed using the Yamane technique for all cases as described here. The CT Lucia 602 is a monofocal aspheric multipiece posterior chamber lens. It is made from hydrophobic polyvinylidene fluoride (PVDF), which is capable of being folded prior to insertion. The lens measures 13.0 mm in overall length, 6.0 mm in optic diameter, and features 5º of haptic angulation. A toric IOL marker was used to mark the limbus at two points, 180º apart. A caliper was then used to mark the eye 2 mm posterior to the limbus, with additional markings created 2 mm temporally and nasally in the superior and inferior directions, respectively, to ensure proper scleral tunnel length. The second marks were 2.5 mm from the limbus, ensuring symmetric tunnels that were tangentially oriented from the limbus. Scleral tunnels were made with 30-gauge TSK needles. The IOL was inserted, both haptics were engaged in the needles, and the haptics were externalized through the scleral tunnels. Flanges were created using external, high-temperature cautery.

Data regarding patients' age, sex, eye laterality, preoperative ophthalmic conditions, preoperative and postoperative visual acuity (VA), axial length (AL), white-to-white (WTW), CT Lucia 602 IOL power, and follow-up time were collected. The lot number of each fractured CT Lucia 602 IOL was obtained.

**Figure 1:**
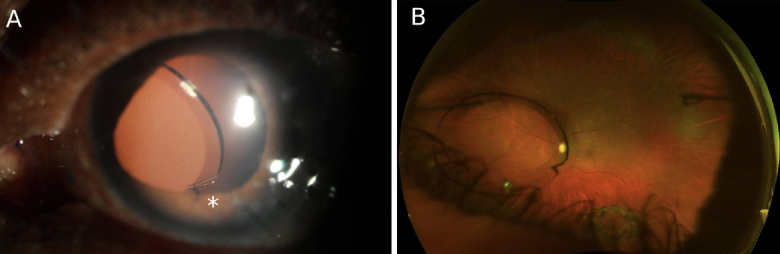
(A) Slit lamp photo highlighting the broken optic (white asterisk) from a disengaged haptic-optic junction. (B) Optos photo with the IOL suspended in the vitreous by the trailing haptic after optic breakage. The leading haptic is seen on the right and remains attached to the sclera.

### Case Description

#### Cases 1 & 2 

The first patient was a 66-year-old man who was left aphakic after cataract surgery and had developed a giant retinal tear with migration of a lens fragment into the subretinal space, which required a pars plana vitrectomy (PPV) and silicone oil placement. The patient was subsequently referred for secondary IOL fixation after removal of the silicone oil. VA was counting fingers preoperatively, which improved to 20/80 with a +10.00 loose lens. After Yamane fixation with a +16.50 diopter (D) CT Lucia, a slight superior IOL tilt was noted on postoperative day one (POD1) with an uncorrected VA of 20/150. By postoperative week 1 (POW1), the leading optic had broken at the haptic-optic junction [Figure 1A], causing the optic to be posteriorly suspended in the vitreous cavity by the trailing haptic [Figure 1B]. Both haptic flanges remained secured to the sclera. The patient denied any history of trauma or vigorous exercise during this period. The patient was scheduled to have a Yamane exchange to replace the broken lens.

**Figure 2:**
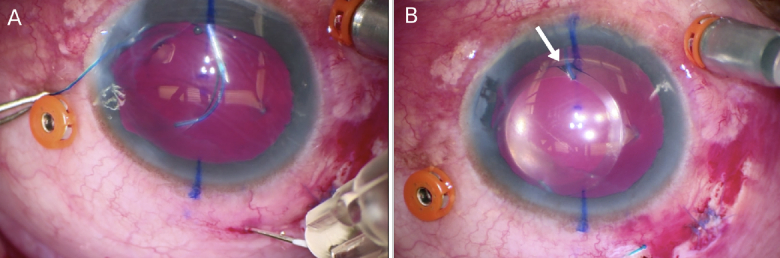
(A) Intraoperative photo showing the leading haptic being fed into the TSK needle with the trailing haptic outside the eye. The optic and haptic-optic junction appears normal and intact. (B) Intraoperative photo taken seconds after the trailing flange had been secured inferiorly. There is now a break in the optic near the leading haptic-optic junction (white arrow) that was not previously seen.

The second patient was a 64-year-old man who presented with a dislocated crystalline lens in the vitreous after suffering blunt trauma two years prior. The patient was scheduled for a PPV to retrieve the dislocated crystalline lens with secondary IOL placement. Preoperative VA was counting fingers with improvement to 20/200 using a +9.00 loose lens. A CT Lucia +17.50 D IOL was placed using the Yamane technique. After both flanges were created, it was intraoperatively noted that the optic near the leading haptic showed a progressive fracture, and evidence of haptic-optic disengagement was imminent [Figure 2A & 2B]. The IOL was subsequently explanted, and the patient received an iris-fixated IOL.

#### Cases 3–5

**Figure 3:**
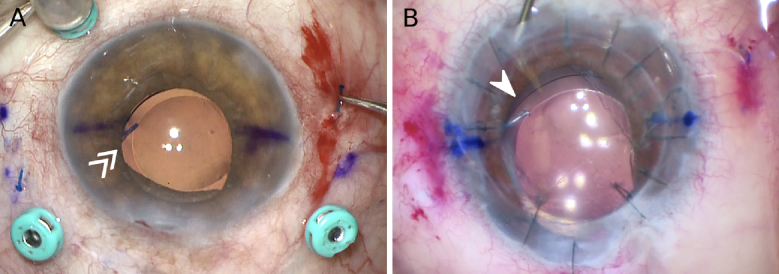
(A) Intraoperative photo of the optic microfracture (white double arrow) from Case 4 is highlighted. (B) Intraoperative photo of the optic microfracture (white arrowhead) from Case 5. All microfractures occurred at the leading haptic. No postoperative complications were noted.

After noting the fractures in the above cases, Yamane intrascleral IOL fixated cases performed by the same surgeon between January 2021 and December 2022 were reviewed. Three cases of optic microfracture at the haptic-optic junction were discovered [Figure 3]. Pertinent information regarding these cases, as well as demographics and characteristics from cases 1 and 2, has been listed in Table 1. No further IOL complications were noted postoperatively in any of the microfracture cases, and these patients did not require any surgical revision.

**Table 1:** Patient and CT Lucia 602 IOL characteristics of fractures and microfractures

**Case no.**	**Age (yrs)**	**Gender**	**Laterality**	**CT Lucia 602 IOL Power (D)**	**AL (mm)**	**IOL lot number**	**WTW (mm)**	**Optic outcome at the haptic-optic junction**	**Length of follow-up**
1	66	M	OS	+16.50	25.73	3S100170165	13.1	Breakage	1 month
2	64	M	OS	+17.50	24.55	3S905320023	12.1	Breakage	4 months
3	82	F	OS	+21.50	22.93	3S2102620298	11.4	Microfracture	3 months
4	44	M	OD	+22.50	23.30	3S2011760138	13.3	Microfracture	13 months
5	66	M	OD	+18.00	30.08	3S014030882	12.9	Microfracture	12 months
AL, axial length; IOL, intraocular lens; WTW, white-to-white; yrs, years; D, diopter; mm, millimeter									

##  DISCUSSION

We presented two cases of optic fracture at the haptic-optic junction while using the Yamane technique, as well as three additional cases of optic microfracture that did not result in frank detachment of the haptic from the optic. All five cases occurred at the haptic-optic junction of the leading haptic. The two optic breakages occurred either intraoperatively or in the early postoperative period. Of the three cases with microfractures, one was noted intraoperatively but was deemed insignificant enough to have no impact on the lens's stability. The other two were identified upon review of past surgical footage. All three were observed without any long-term complications.

Secondary IOL implantations are known to have potential complications. In the original article from Yamane et al, the authors noted iris capture in 8%, vitreous hemorrhage in 5%, elevation of the IOL in 3%, hypotony in 2%, and cystoid macular edema (CME) in 1% of patients.^[1]^ These complications are nonspecific and can also be seen in other secondary IOL fixation methods. On the other hand, lens tilt and lens decentralization are occurrences somewhat specific to the flanged intrascleral IOL fixation technique, which can occur if the needle insertions are not 180º apart or if the scleral tunnels vary in length or direction; however, these complications are rare. A case series by Fan et al of 19 patients who underwent IOL fixation through the Yamane technique revealed minimal complications, with only one case of IOL tilt that did not require additional procedures and one retinal tear.^[3]^ Transient complications in this case series included vitreous hemorrhage in 15% and CME in 15% of patients. Muth et al investigated the safety of different intrascleral fixation techniques, including GoreTex suture, Prolene suture, and Yamane technique.^[4]^ The study showed that the Yamane technique had the lowest revision rate of the three groups, with only 4.5% of cases requiring IOL refixation. Overall, the Yamane technique is considered a safe and efficient method with uncommon complications.

Rare case reports of spontaneous haptic-optic disinsertion of various three-piece IOLs ranging from the immediate postoperative period to years after implantation have been described in the literature.^[5,6,7,8]^ However, in all of those cases, posterior chamber polymethyl methacrylate (PMMA) IOLs have been placed with various possible disinsertion reasons such as manufacturing error, possible poor capsular bag fixation, undetected trauma at the time of IOL insertion, and haptic-optic junction fatigue over time.

One of the IOLs most used with the Yamane technique is MA60AC (Alcon, Fort Worth, TX, USA). It has an anterior asymmetric biconvex 6.0 mm optic. However, this multipiece lens, with an overall length of 13.0 mm, features PMMA haptics angled at 10º, compared to the CT Lucia, which is made of PVDF haptics with a 5 ${}^{\circ }$ angulation. The cases described herein were performed by the same experienced surgeon and utilized the CT Lucia 602 IOL, rather than PMMA IOLs. The haptic arms of the CT Lucia 602 are made of PVDF, which is more resilient and easier to manipulate than haptics made of PMMA. However, the force required to avulse the haptic from the optic is less clear. Some studies have shown that the force needed to remove the haptic from the sclera and the force for haptic-optic breakage are significantly higher for PVDF IOLs compared to PMMA IOLs.^[9]^ A biomechanical study conducted by Ma et al comparing the CT Lucia 602 to the ZA9003 (Johnson & Johnson, Santa Ana, California, USA), LI61AO (Bausch & Lomb, Rochester, New York, USA), and MA60AC (Alcon Laboratories, Fort Worth, Texas, USA) IOLs demonstrated the force required to break the haptic from the sclera and the haptic-optic disinsertion force was greatest for the CT Lucia.^[9]^ However, while the ZA9003 and MA60AC had significantly higher haptic-optic disinsertion forces compared to their scleral dislocation forces, there were no significant differences between the sclera-haptic and haptic-optic disinsertion forces of the CT Lucia 602 when using 30G TSK needle scleral tunnels.

Interestingly, Cheng et al reported haptic-related complications using the MA60AC IOL with various sutureless techniques.^[10]^ They suggested that different scleral fixation techniques present varying risks of complications. In our series, the same surgical technique without any variations or excessive haptic handling was implemented. Multiple studies comparing the PMMA (MA60AC) and PVDF (CT Lucia) IOLs showed the significance of suture selection and flange characteristics for the flanged intrascleral fixation technique.^[11,12]^ Yannuzi et al also demonstrated that while the CT Lucia required 2.8 times greater force for haptic breakage than the PMMA MA60N (Alcon Laboratories, Fort Worth, Texas, USA) IOL, the haptic-optic avulsion force for the CT Lucia 602 was statistically weaker than that of the MA60N.^[13]^ This latter finding contradicts that of Ma et al, which found the CT Lucia 602 to require the greatest haptic-optic disinsertion force when compared to other PMMA lenses. This discrepancy could be because Yannuzi et al found only a mean difference of 0.25 newtons between the PMMA MA60N and PVDF CT Lucia's haptic-optic disinsertion force. While statistically significant, this slight raw difference is unlikely to hold clinical significance, as acknowledged by the investigators.^[13]^ In our cases, the optic was sheared and fractured near the haptic-optic junction rather than a disinsertion of the haptic. Given these prior studies, we speculate that the junction may be an intrinsically weaker point in the CT Lucia IOL and may have contributed to the optic breakages and microfractures due to torquing forces.

Possible etiologies for excessive torquing forces include stretch on the IOL due to a large WTW, a misdirected angle of the scleral tunnels, or a posteriorly fixated IOL creating stretch across the haptics.^[14]^ We believe the latter two causes to be less likely, given that tunnel measurements were performed meticulously and consistently across all cases, and numerous cases have been successful in the past without any optic breakages. Nevertheless, we cannot definitively rule out any unintentional scleral separation during creation of the tunnels that may have caused asymmetry in the scleral tunnels. Compared to the normative data derived from the literature, three patients in our case series (cases 1, 4, and 5) had a WTW > 2 standard deviations (SD) from the reported average, and one patient (case 5) had an AL > 2 SDs.^[14,15]^ Large WTW and long AL have been shown to cause more IOL tilt and decentralization in previous studies.^[16,17]^ While no studies have been performed on the effect of WTW and AL on scleral-fixated IOLs, we hypothesize that these larger parameters may cause the haptics to be stretched and the angle of insertion at the haptic-optic junction to be distorted. While this could account for optic breakage in case 1 and microfractures in cases 4 and 5, the WTWs and ALs of the other patients were within one standard deviation from average values, and thus cannot be the sole explanation.

The etiology of our optic fractures and microfractures at the haptic-optic junction is largely unclear. All cases were performed by the same experienced surgeon using identical technique. The sclerotomy sites were carefully measured and delineated with calipers, and the scleral tunnel lengths were of equal length and orientation. All five of the CT Lucia 602 IOLs had different lot numbers, indicating they did not come from the same manufacturing batches. We speculate that the cause is an inherent weakness in the IOLs' haptic-optic integrity, rather than a manufacturing error. As it was the leading haptic that fractured in all cases, the forces required to manipulate the IOL into position likely play a role in this process. The two cases of optic breakages occurred within a month of each other, which suggests that there may have been an overall change in the way the CT Lucia 602 IOLs are designed, making them less resistant to the manipulation required with the Yamane technique. The haptic-optic design is proprietary information and unavailable for analysis.

In summary, our cases represent a rare complication of CT Lucia 602 IOLs placed using the Yamane technique. Given that one out of two haptic-optic breakages did not occur immediately, but rather one week after the initial surgery, we recommend that patients with CT Lucia 602 IOL implantation using the Yamane technique undergo close follow-up to facilitate prompt identification of this complication. While we hypothesize that the haptic-optic breakage is due to an inherent problem with the IOL, it is essential to be aware of variable preoperative measurements and to employ careful surgical technique to minimize further complications.

##  Financial Support and Sponsorship

None.

##  Conflicts of Interest

None.
